# Risk factors of extrauterine growth restriction in very preterm infants with bronchopulmonary dysplasia: a multi-center study in China

**DOI:** 10.1186/s12887-022-03405-z

**Published:** 2022-06-24

**Authors:** Lian Wang, Xin-Zhu Lin, Wei Shen, Fan Wu, Jian Mao, Ling Liu, Yan-Mei Chang, Rong Zhang, Xiu-Zhen Ye, Yin-Ping Qiu, Li Ma, Rui Cheng, Hui Wu, Dong-Mei Chen, Ling Chen, Ping Xu, Hua Mei, San-Nan Wang, Fa-Lin Xu, Rong Ju, Zhi Zheng, Xiao-Mei Tong, the Chinese Multicenter EUGR Collaborative Group, Lian Wang, Xin-Zhu Lin, Wei Shen, Zhi Zheng, Fan Wu, Jian Mao, Ling Liu, Yan-Mei Chang, Xiao-Mei Tong, Rong Zhang, Xiu-Zhen Ye, Yin-Ping Qiu, Li Ma, Rui Cheng, Hui Wu, Dong-Mei Chen, Ling Chen, Ping Xu, Hua Mei, San-Nan Wang, Fa-Lin Xu, Rong Ju, Gui-Nan Li, Long Li, Zhe Zhang, Fei Bei, Chun Deng, Ping Su, Ling-Ying Luo, Xiao-Hong Liu, Li-Jun Wang, Shu-Qun Yu

**Affiliations:** 1 Department of Neonatology, Women and Children’s Hospital, School of Medicine, Xiamen University, Xiamen, China; 2 Xiamen Key Laboratory of Perinatal-Neonatal Infection, Xiamen, China; 3 Department of Neonatology, The Third Affiliated Hospital of Guangzhou Medical University, Guangzhou, China; 4 Department of Pediatrics, Shengjing Hospital of China Medical University, Shenyang, China; 5 Department of Neonatology, Guiyang Maternal and Child Health Hospital·Guiyang Children’s Hospital, Guiyang, China; 6 Department of Pediatrics, Peking University Third Hospital, Beijing, China; 7 Department of Neonatology, Children’s Hospital of Fudan University, Shanghai, China; 8 Department of Neonatology, Guangdong Province Maternal and Children’s Hospital, Guangzhou, China; 9 Department of Neonatology, General Hospital of Ningxia Medical University, Yinchuan, China; 10 Department of Neonatology, Children’s Hospital of Hebei Province, Shijiazhuang, China; 11 Department of Neonatology, Children’ Hospital of Nanjing Medical University, Nanjing, China; 12 Department of Neonatology, The First Hospital of Jilin University, Changchun, China; 13 Department of Neonatology, Quanzhou Maternity and Children’s Hospital, Quanzhou, China; 14 Department of Pediatrics, Tongji Hospital, Tongji Medical College, Huazhong University of Science and Technology, Wuhan, China; 15 Department of Neonatology, Liaocheng People’s Hospital, Liaocheng, China; 16 Department of Neonatology, the Affiliate Hospital of Inner Mongolia Medical University, Hohhot, China; 17 Department of Neonatology, Suzhou Municipal Hospital, Suzhou, China; 18 Department of Neonatology, The Third Affiliated Hospital of Zhengzhou University, Zhengzhou, China; 19 Department of Neonatology, Chengdu Women’ and Children’s Central Hospital, School of Medicine, University of Electronic Science and Technology of China, Chengdu, China

**Keywords:** Bronchopulmonary dysplasia, Very preterm infants, Perinatal nutrition, Extrauterine growth restriction

## Abstract

**Objective:**

Nutritional deficiency soon after birth is a risk factor of chronic lung disease (bronchopulmonary dysplasia, BPD). Afflicted infants are further prone to inadequate growth during hospitalization (extrauterine growth restriction, EUGR). This multi-center retrospective study investigated risk factors of EUGR, specifically in very preterm infants with BPD.

**Method:**

Data of infants with BPD who were born less than 32 weeks gestation (*n* = 1010) were collected from 7 regions of China. All infants were non-small for gestational age at birth. Infants were characterized as EUGR or non-EUGR at 36 weeks gestation or discharge, or stratified by gestational age or birthweight. Logistic regression analysis was applied.

**Results:**

In 65.5% of the population, the BPD was mild. Infants with severe BPD (8.3%) had the highest rate of EUGR (72.6%, *P* < 0.001). Groups stratified by gestational age did not differ in rates of EUGR, but the birthweight of the EUGR group was significantly lower than that of the non-EUGR (*P* < 0.001). Birthweights of < 1000, 1000–1499, and ≥ 1500 g showed EUGR rates of 65.9%, 43.4%, and 23.8%, respectively (*P* < 0.001). Overall, the independent risk factors of EUGR were: moderate-to-severe BPD, gestational hypertension, cesarean section, cumulative fasting time, time required to achieve 110 kcal/kg/d, and hemodynamically significant patent ductus arteriosus (hsPDA).

**Conclusion:**

In very preterm infants with BPD, the lower the birthweight or the more severe the BPD, the greater the risk of EUGR. In those with hsPDA, or moderate-to-severe BPD, it is especially important to prevent EUGR through perinatal management, enteral nutrition, and nutritional strategies.

## Background

Bronchopulmonary dysplasia (BPD) is a chronic lung disease and a common complication of very preterm infants (i.e., premature infants born younger than 32 weeks gestational age). Early nutritional deficiency after birth is an independent risk factor of BPD [1]. Inadequate management of nutrition can alter the normal development of the lungs and reduce resistance to hypoxia, mechanical ventilation, and infectious diseases, slowing the repair of lung injury, and worsening BPD. In a vicious cycle, malnutrition is aggravated by mechanical ventilation, fluid restriction, diuretics, and postnatal glucocorticoid. Thus, during hospitalization infants with BPD are at high risk for postnatal growth failure or inadequate growth, which is known as extrauterine growth restriction (EUGR) [2].

The energy requirements of very premature infants with BPD are above normal due to increased respiratory demand and chronic lung injury. The intrauterine storage of these infants was insufficient and they are prone to negative nitrogen balance. This makes it difficult to meet their excess energy demand, furthering the risk of EUGR [3]. One study reported that infants with BPD at discharge were significantly more likely to display EUGR, compared with infants without BPD. The authors concluded that infants with BPD are more susceptible to EUGR. Furthermore, at ages corrected at 3 months, the percentage of preterm babies with BPD at discharge who had achieved at least the minimum appropriate birthweight (P_10_) was significantly lower than that of the control group without BPD, among other morbidities and slowed motor development [4].

Small for gestational age (SGA) refers to a birthweight lower than the tenth percentile (< P_10_) of the average birthweight of infants of the same gender and gestational age. In very premature infants, being SGA is an important prenatal risk factor for BPD, and an independent risk factor for EUGR [5, 6]. However, postnatal growth restriction in infants who are SGA is probably not of postnatal origin, but rather an ongoing process from before birth. Figueras-Aloy et al. [7] suggested the concept “true-EUGR,” referring to EUGR in infants who are not SGA, and in whom EUGR is due to factors other than fetal growth impairment. Therefore, they recommend that in studies of prevention or trials testing treatments or nutrition regimens for EUGR, the preterm subsample who are SGA should be clearly differentiated from the non-SGA.

In recent years, more and more studies have focused on the nutritional management of preterm infants with BPD in China. However, there have been limited studies on EUGR specifically in very preterm infants with BPD who are not SGA, and in China no national multi-center research has been conducted.

The present retrospective multicenter study from China determined the incidence of EUGR specifically in very preterm infants with BPD who were not-SGA (NSGA), and investigated risk factors of EUGR. The study provides an evidence-based medical reference for optimizing nosocomial nutrition and improving the short- and long-term prognoses of these patients.

## Methods

The research protocol was approved by the Ethics Committee of Women and Children’s Hospital affiliated to Xiamen University/Xiamen Maternal and Child Health Hospital (Batch number kY-2019–016).

### Research subjects

The Chinese Multicenter EUGR Collaborative Group was founded in 2019, to investigate the incidence and related factors of EUGR in very preterm infants during hospitalization in different regions of China (Trial registration: chictr.org.cn, number: ChiCTR1900023418). The clinical data were prospectively recorded at 28 hospitals in 7 regions of China from September 2019 to December 2020. The data regarding NSGA-very preterm infants with BPD (*n* = 1010) were retrospectively analyzed for the present report. At corrected gestational age 36 weeks or at discharge, the study population was stratified as having EUGR or not having EUGR, with 450 and 560 very preterm infants in the EUGR and non-EUGR groups, respectively.

For inclusion in this study, all the subjects conformed to these criteria: gestational age < 32 wk; diagnosis of BPD; hospitalization ≥ 28 days; hospital admission within 24 h after birth; and NSGA at birth. Potential subjects with any of the following were excluded: congenital malformation or genetic metabolic disease; death during hospitalization; interruption of treatment; automatic discharge; or incomplete data.

The criteria for hospital discharge were the following: cure of the primary disease; stable vital signs (discharge with oxygen was allowed); milk volume reached total enteral feeding; and corrected gestational age ≥ 36 weeks.

### Data collection

The collected data included the following: maternal complications during pregnancy; general clinical data of preterm infants and nutritional status during hospitalization; complications; and major treatments. Perinatal data consisted of gestational age at birth; birthweight; gender; delivery mode; One-minute Apgar score; full course of prenatal glucocorticoid use; and maternal pregnancy complications such as gestational hypertension and diabetes. Indicators related to infant growth and nutrition were: greatest weight loss; days to regain birthweight; growth velocity after birthweight was regained; start of enteral nutrition; age when total enteral nutrition was reached; duration of parenteral nutrition; cumulative fasting days; accumulated calories during the first week of hospitalization; accumulative doses of amino acid and fat emulsions during the first week of hospitalization; and days to reach 110 kcal/kg/d total calorie intake and 110 kcal/kg/d oral calorie intake.

Primary complications during hospitalization were the following: neonatal respiratory distress syndrome; hemodynamically significant patent ductus arteriosus (hsPDA); feeding intolerance; anemia requiring blood transfusion; early- and late-onset sepsis; neonatal necrotizing enterocolitis Bell stage ≥ 2 (see below); intraventricular hemorrhage (IVH) grade III-IV (see below); periventricular leukomalacia; retinopathy of prematurity; metabolic bone disease of prematurity (MBDP); and parenteral nutrition-associated cholestasis. Also considered for analysis were these main treatments: postnatal glucocorticoid therapy; duration of invasive mechanical ventilation; and total hospitalization.

### Definitions and diagnostic criteria

SGA was defined as a birthweight lower than the tenth percentile (< P_10_) of the average birthweight of infants of the same gender and gestational age. A diagnosis of BPD was indicated by at least 28 days of oxygen therapy above the fraction of inspired oxygen (FiO_2_) of 0.21. With reference to the FiO_2_ requirement at 36 weeks post-menstruation age or discharge, whichever came first, BPD was characterized as mild (room air); moderate (FiO_2_ < 0.3), or severe (FiO_2_ ≥ 0.3 or requiring positive pressure ventilation) [8]. Referring to Fenton 2013 [9], EUGR was defined as the tenth percentile of weight below the growth curve at the corrected gestational age of 36 weeks or at discharge.

A hsPDA was considered a patent ductus arteriosus catheter diameter > 1.5 mm, left atrial diameter/aortic diameter ≥ 1.4, and left-to-right shunt (or bi-directional bi-phase shunt). In addition, the patent ductus arteriosus was accompanied by at least one of these following clinical manifestations: heart murmur; tachycardia; increased respiration; increased pulse pressure; hypotension; flushing; or cardiac dilation [10]. Retinopathy of prematurity was considered interventional when intravitreal drug injection, laser therapy, or surgery were required. The diagnostic criteria for early- and late-onset sepsis refers to the Expert Consensus on the Diagnosis and Management of Neonatal Sepsis (version 2019) [11]. NEC was defined as Bell stage ≥ 2 [12]; IVH grade III-IV was IVH with ventricular enlargement or parenchymal hemorrhage, as classified according to the Papile system [13]; MBDP was considered serum alkaline phosphatase > 900 IU/L, and serum phosphorus < 1.8 mmol/L [14]. The days to reach total enteral nutrition was the time required for oral feeding of milk to reach 150 mL/kg/d [15]. Growth velocity after birthweight was regained (g/kg/d) was calculated as: [1000 × ln (Wn / W1)] / [Dn – D1], where Wn and W1 are the weights at discharge and birth, respectively, and Dn and D1 are the total days of hospital stay and days to regain birthweight [16]. The following diagnoses referenced Practical Neonatology (Fifth Edition) [17]: respiratory distress syndrome; feeding intolerance; periventricular leukomalacia; parenteral nutrition-associated cholestasis; and anemia.

### Statistical methods

All data were processed using SPSS 23.0 statistical software. The counting data are shown as number of cases and percentage. The chi-squared test or Fisher’s exact test was used for comparisons between groups. Non-normally distributed quantitative data are shown as median and interquartile spacing (M [Q1, Q3]), and the Mann–Whitney U test was used for comparisons between groups. Pairwise comparisons between multiple groups were performed by Bonferroni test. Disease risk factors were analyzed by logistic regression. *P* < 0.05 was considered statistically significant.

## Results

### EUGR at different levels of BPD, gestational age, and birthweight

The study population comprised 1010 NSGA-very preterm infants with BPD. The rate of EUGR was 44.6% (450/1010). When the population was stratified by BPD severity, then mild, moderate, and severe BPD accounted for 65.5% (661/1010), 26.2% (265/1010) and 8.3% (84/1010) of the overall population; the respective rates of EUGR were 38.6% (255/661), 50.6% (134/265), and 72.6% (61/84). The percentage of patients with EUGR was significantly higher in the moderate and severe BPD groups compared with the mild BPD group (*P* < 0.001). The more severe the BPD, the higher the rate of EUGR.

When the overall population was stratified by EUGR (Table 1), the mean gestational age of the EUGR group (29.0 [27.8, 30.3]) was comparable to that of the non-EUGR group (29.1 [28.0, 30.0]; Z = –0.055, *P* = 0.956). There was no significant difference in the rates of EUGR among the gestational age stratifications (< 28, 28–29 + ^6^, 30–31 + ^6^; *P* = 0.097). The mean birthweight of the EUGR group (1145 g [990, 1300]) was significantly lower than that of the non-EUGR group (1300 g [1100, 1450]; Z = –9.031, *P* < 0.001). By the birthweight stratifications < 1000 g, 1000–1499 g, and ≥ 1500 g, the corresponding rates of EUGR were 65.9% (118/179), 43.4% (297/684), and 23.8% (35/147). Thus, the lower the birthweight, the higher the rate of EUGR (*P* < 0.001).

**Table 1:** EUGR at different levels of BPD, gestational age, and birthweight

		Number(n)	Proportion(%)	EUGR[n(%)]	*Z*	*P*
Subjects, n		1010	—	450	—	—
Severity of BPD	Mild	661	65.5	255 (38.6)	40.216	< 0.001
	Moderate^a^	265	26.2	134 (50.6)		
	Severe^a,b^	84	8.3	61 (72.6)		
Gestational age, wk	< 28	258	25.5	122 (47.3)	4.671	0.097
	28–29^+6^	451	44.7	184 (40.8)		
	30–31^+6^	301	29.8	144 (47.8)		
Birthweight, g	< 1000	179	17.7	118 (65.9)	59.046	< 0.001
	1000–1499^c^	684	67.7	297 (43.4)		
	≥ 1500^c,d^	147	14.6	35 (23.8)		

^a^*P* < 0.0125 cf. the baseline mild BPD group; ^b^*P* < 0.0125 cf. the baseline moderate BPD group; ^c^*P* < 0.0125 cf. the baseline birthweight < 1000 g group; ^d^*P* < 0.0125 cf. the baseline birthweight 1000–1499 g group

### Obstetrical and neonatal characteristics

The rates of cesarean delivery, One-minute Apgar score ≤ 7, and gestational hypertension were each higher in the EUGR group than that of the non-EUGR group (Table 2). There were no significant differences in gender, prenatal glucocorticoid use, or rate of gestational diabetes between the 2 groups.

**Table 2:** Obstetrical and neonatal characteristics^a^

	EUGR	Non–EUGR	*Z*	*P*
Subjects, n	450	560	—	—
Prenatal GC	342 (76.0)	445 (79.5)	1.740	0.187
Male	263 (58.4)	335 (59.8)	0.196	0.658
Cesarean delivery	266 (59.1)	271 (48.4)	11.512	0.001
One-minute Apgar ≤ 7	234 (52.0)	217 (38.8)	17.724	< 0.001
Gestational diabetes	87 (19.3)	113 (20.2)	0.112	0.738
Gestational hypertension	105 (23.3)	48 (8.6)	42.299	< 0.001

^a^Reported as n (%)

*GC* Glucocorticoid

### Complications and main treatments

The results of the univariate analysis regarding complications during hospitalization showed that compared with the non-EUGR group, the patients with EUGR had significantly higher rates of the following (Table 3): hsPDA, anemia requiring blood transfusion, feeding intolerance, MDBP, late-onset sepsis, parenteral nutrition-associated cholestasis, and interventional retinopathy of prematurity (all, *P* ≤ 0.001). The rate of postnatal glucocorticoid therapy was also significantly higher in the EUGR group than the non-EUGR (*P* = 0.002), as were days of invasive mechanical ventilation, and total hospital stay (*P* < 0.001). The EUGR and non-EUGR groups were statistically similar regarding rates of respiratory distress syndrome, neonatal necrotizing enterocolitis Bell stage ≥ 2, early-onset sepsis, IVH grade III-IV, and periventricular leukomalacia.

**Table 3:** Complications and main treatments^a^

	EUGR	Non–EUGR	*Z*	*P*
Subjects, n	450	560	—	—
NRDS	374 (83.1)	440 (78.6)	3.288	0.070
Postnatal glucocorticoid therapy	153 (34.0)	141 (25.2)	9.409	0.002
hsPDA	144 (32.0)	124 (22.1)	12.436	< 0.001
Interventional ROP	39 (8.7)	20 (3.6)	11.777	0.001
Anemia requires blood transfusion	386 (85.8)	418 (74.6)	19.053	< 0.001
Feeding intolerance	218 (48.4)	195 (34.8)	19.158	< 0.001
NEC Bell stage ≥ 2	40 (8.9)	52 (9.3)	0.047	0.828
MBDP	23 (5.1)	15 (2.7)	4.077	0.043
IVH grade III–IV	12 (2.7)	16 (2.9)	0.034	0.855
Periventricular leukomalacia	24 (5.3)	18 (3.2)	2.811	0.094
Early–onset sepsis	84 (18.7)	106 (18.9)	0.011	0.916
Late–onset sepsis	105 (23.3)	74 (13.2)	17.521	< 0.001
PNAC	88 (19.6)	61 (10.9)	14.888	< 0.001
Invasive mechanical ventilation, d^b^	3 (0, 10)	1 (0, 5)	–6.400	< 0.001
Total hospital stay, d ^b^	62 (50, 75)	53 (45, 66)	–6.553	< 0.001

^a^Reported as n (%), unless indicated otherwise; ^b^[M (Q1, Q3)]

*hsPDA* Hemodynamically significant patent ductus arteriosus, *IVH* Intraventricular hemorrhage, *NEC* Neonatal necrotizing enterocolitis, *NRDS* Neonatal respiratory distress syndrome, *MBDP* Metabolic bone disease of prematurity, *PNAC* Parenteral nutrition-associated cholestasis, *ROP* Retinopathy of prematurity

### Growth and nutrition-related indicators

Compared with the non-EUGR group, the EUGR group showed significantly slower growth velocity. The EUGR group experienced a longer period of cumulative fasting, began enteral nutrition later, and attained total enteral nutrition later; and the duration of parenteral nutrition was also longer (all, *P* < 0.001; Table 4). The EUGR infants also required a longer time to achieve target total calorie intake (110 kcal/kg) and 110 kcal/kg oral calories (both, *P* < 0.001), and time to regain birthweight (*P* = 0.026). The accumulative doses of fat emulsions given to the EUGR group during the first week were significantly higher than that of the non-EUGR group, but the accumulated calories were less (both, *P* = 0.003). The 2 groups were similar regarding the accumulative doses of amino acids during the first week, and the percentage of greatest weight loss at discharge.

**Table 4:** Growth and nutrition-related indicators ^a^

	EUGR	Non–EUGR	*Z*	*P*
Subjects, n	450	560	—	—
Time to start of enteral nutrition, h	26 (15, 62)	21 (10, 36)	–5.192	< 0.001
Total enteral nutrition, d	33 (24, 45)	28 (20, 39)	–5.664	< 0.001
Duration of parenteral nutrition, d	29 (20, 40)	22 (15, 33)	–7.646	< 0.001
Cumulative fasting, d	3.8 (1.0, 7.2)	1.9 (0.8, 4.0)	–7.783	< 0.001
Accumulative amino acids, g/kg ^b^	17.0 (14.1, 19.0)	16.7 (14.3, 18.9)	–1.389	0.165
Accumulative fat emulsions, g/kg ^b^	13.4 (11.0, 15.7)	13.0 (10.5, 15.0)	–3.002	0.003
Accumulated calories, kcal/kg ^b^	487 (414, 551)	500 (435, 571)	–2.995	0.003
Total calories up to 110 kcal/kg/d, d	11 (7, 18)	8 (7, 14)	–5.283	< 0.001
Oral calories up to 110 kcal/kg/d, d	30 (22, 43)	24 (17, 33)	–7.565	< 0.001
Greatest weight loss %	7.0 (3.6, 10.0)	6.3 (3.7, 9.2)	–1.395	0.163
Regain birthweight, d	10 (7, 12)	9 (7, 12)	–2.226	0.026
Growth velocity, g/kg/d	12.8 (11.3, 14.2)	14.9 (13.3, 16.3)	–12.478	< 0.001

^a^Reported as M (Q1, Q3); ^b^during the first week

### Risk factors of EUGR

The variables with statistical significance in the univariate analysis (Tables 2, 3 and 4) and severity of BPD were included in the multivariate logistic regression analysis (Table 5), which showed that the following were independent risk factors for EUGR: gestational hypertension, cesarean section, long fasting time, late attainment of 110 kcal/kg/d of oral calories, hsPDA, and moderate or severe BPD.

**Table 5:** Multivariate logistic regression analysis of the risk factors of EUGR

	β	SE	Wald χ^2^	*P*–value	OR (95% CI)
Gestational hypertension	1.118	0.205	29.891	< 0.001	3.060 (2.049–4.568)
Cesarean delivery	0.407	0.146	7.790	0.005	1.502 (1.129–1.999)
Cumulative fasting days	0.070	0.018	15.629	< 0.001	1.072 (1.036–1.110)
Days to 110 kcal/kg/d oral calories	0.025	0.006	18.951	< 0.001	1.025 (1.014–1.036)
HsPDA	0.179	0.079	5.165	0.023	1.196 (1.025–1.396)
BPD^a^	—	—	16.572	< 0.001	—
Moderate BPD	0.328	0.158	4.292	0.038	1.388 (1.018–1.893)
Severe BPD	1.068	0.278	14.734	< 0.001	2.911 (1.687–5.023)

^a^The reference category for setting dummy variables is mild BPD

## Discussion

This retrospective study analyzed evidence of the postnatal growth patterns of a large general population of very preterm infants with BPD, NSGA, treated at several regional medical centers in China. Postnatal growth retardation (EUGR) in SGA infants may be a continuation of the intrauterine process previously affecting fetal growth. The influencing factors of EUGR occurring at the time of discharge but not SGA at birth may be more closely related to postpartum factors. Figueras-Aloy et al. [7] showed that among 479 very-preterm infants, EUGR occurred in 50.7% at 34-36 postmenstrual weeks and 21.1% at 2-2.5 years, but among 411 non-SGA infants, these figures were 42.7% and 15.4%, respectively. Therefore, we clearly differentiated SGA and non-SGA to explore the real risk factors for EUGR in very preterm infants with BPD. In this population of NSGA-very preterm infants with BPD, growth was affected by the severity of BPD, pregnancy-related etiologies, postnatal enteral nutrition, and the presence of hsPDA.

The rates of EUGR were 38.6%, 50.6% and 72.6%, respectively, for infants with mild, moderate, and severe BPD. Thus, as BPD increased in severity, so too did the likelihood of EUGR. Furthermore, moderate or severe BPD were closely related to the occurrence of EUGR and were independent risk factors. This is in accord with the findings of Natarajan et al. [18], who investigated the outcomes of 375 extremely premature infants with severe BPD and observed that, at 36 weeks post-menstrual age, 53% suffered EUGR. Another multi-center study in China found that moderate-to-severe BPD was an independent risk factor for EUGR in NSGA infants (OR = 2.241, 95% CI: 1.173–281) [19].

The present study also determined that, when the population was stratified by birthweight, the percentage with EUGR increased with each lower birthweight level. However, the gestational ages of the EUGR and non-EUGR groups were comparable, and there was also no significant difference in the rates of EUGR among gestational age stratifications. This is consistent with a multi-center investigation on the nutritional status of premature infants in the Neonatal Intensive Care Unit conducted by the Research Group for the Nutrition of Premature Infants in China [20]. Another study of 225 very preterm infants with BPD found similar results—the average birth weight and birth weight as a percentile of the Fenton curve in the EUGR group were lower than that of the non-EUGR group, but there was no significant difference in the average gestational age between the two groups [21]. These results suggest that EUGR is more closely related to birth weight than gestational age in very preterm infants with BPD. Premature infants with lower birth weight have poorer organ development and higher possibility of feeding intolerance and serious diseases, resulting in insufficient nutritional intake and high energy metabolism consumption, thus increasing the risk of EUGR.

In the present study of NSGA-very preterm infants with BPD, the multivariate analysis showed that gestational hypertension and cesarean section were independent risk factors for EUGR. A multicenter prospective cohort study of very premature infants reported that the rate of EUGR in premature infants with gestational hypertension (50.2%) was significantly higher than that of the control group (25.3%), which also suggested that gestational hypertension was an independent risk factor for EUGR (OR = 1.368, 95% CI: 1.081–1.731; P = 0.009) [22].

Gestational hypertension, especially preeclampsia and eclampsia, may lead to abnormal recasting of the maternal uterine spiral artery and insufficient infiltration of trophoblasts, placental ischemia, and hypoxia. The resultant decrease in placental perfusion will restrict fetal intrauterine growth, making low birthweight (in the lower percentile of the Fenton curve) more likely. Previous studies have reported that the percentile on the Fenton curve at birth is an important predictor of EUGR [23]. In addition, gestational hypertension can increase the need for emergency cesarean section [24], which is an independent risk factor for low Apgar score [25]. These infants are often born sick and even require mechanical ventilation, leading to an increased risk of BPD and EUGR.

The multivariate regression analysis of the present study showed that the number of cumulative fasting days and days to reach 110 kcal/kg/d oral calorie intake were independent risk factors for EUGR. In very premature infants with BPD, many factors contribute to enteral malnutrition. Hemodynamic or respiratory instability after birth can delay the start of enteral nutrition with repeated fasting and slowing milk addition. Theile et al. [26] conducted a retrospective analysis of 88 extremely low birthweight infants with BPD and found that the fewer the days needed to achieve oral calories up to 110 or 120 kcal/kg/d, the higher the percentage with fetal growth rate ≥ 16 g/kg/d. Alshaikh et al. [27] also confirmed that delay in reaching full enteral feed was associated with increased risk of BPD (OR = 1.03; 95% CI: 1.00–1.06), and thereby EUGR.

In our study, significant differences in the infants in the EUGR group included beginning enteral nutrition (26 h) and full enteral feeding (33 d) later, and experiencing longer fasting time (3.8 d), compared with the non-EUGR group (21 h, 28 d, and 1.9 d, respectively). Moreover, our study also showed that the EUGR group had more severe clinical conditions that required longer invasive mechanical ventilation, and a higher rate of complications such as low one-min Apgar score, hsPDA, anemia requiring blood transfusion, feeding intolerance, and late-onset sepsis. All these factors delay decisions regarding nutrition management, which leads to unnecessary fasting, neglect of early enteral feeding, and delay of total enteral nutrition, and results in early insufficient oral calorie intake.

In preterm infants, hsPDA particularly can decrease superior mesenteric artery blood flow and increase the risk of feeding intolerance and IVH. Moreover, hsPDA often requires drug treatment. Clinicians are often concerned that nonsteroidal anti-inflammatory drugs may increase gastrointestinal injury. During drug treatment, they often empirically limit the amount of milk, delay milk addition, and even impose fasting. All these measures will delay the time to reach 110 kcal/kg/d enteral energy intake, thus increasing the risk of EUGR. Mabhandi et al. [28] found that hsPDA is a risk factor for slow postnatal growth velocity (i.e., ≤ 15 g/kg/d) in extremely low birthweight infants.

In a retrospective study [29], infants with diagnosed hsPDA were characterized with low fluid intake, low energy, and low protein intake. In the multivariate analysis, there was a statistically significant association between the presence of hsPDA and weight gain during the first 28 days of life (OR = 3.35, 95% CI:0.71–5.99, *P* = 0.01) [29]. Consistent with the above findings, the present study also showed that hsPDA was an independent risk factor for EUGR in NSGA-very preterm infants with BPD. Therefore, clinicians should pay more attention to enteral nutrition, avoid unnecessary fasting, and bring the infant to full enteral feeding as quickly as possible. At the same time, nutritional management of hsPDA should be optimized to provide adequate oral energy intake, thus reducing the possibility of EUGR.

A limitation of this study is that among the 28 hospitals across the vast territory of China, there were differences in strategies of nutrition management. However, we adhered to the guideline for clinical practice of nutrition support in Chinese neonates (version 2013) [30], and implemented nutrition management accordingly. Thus, these differences in strategies of nutrition management are relatively small, but also objectively reflects the real nutritional status of this part of the Chinese population. Another deficiency is the lack of detailed data regarding factors such as fluid intake in the first week, protein energy ratio, milk adding speed, reasons for fasting, and others. This makes it impossible to comprehensively analyze the nutritional status of this study population.

This is the first multi-center study to focus on the prevalence and risk factors of EUGR among NSGA-very preterm infants with BPD in China. The centers were all grade III and grade A hospitals, with relatively complete data and high reliability. The study provides a basis for better understanding and improving the nutritional status of very premature infants with BPD in China, and optimizing the nutritional support program.

## Conclusions

Very preterm infants with BPD but not small for gestational age remain at high risk of EUGR. We should optimize comprehensive hospital management strategies to improve the short- and long-term prognoses of these infants. Perinatal care should be vigilant to reduce the occurrence of gestational hypertension and need for cesarean section. Enteral nutrition should be active and unnecessary fasting avoided. Adequate oral energy should be provided as soon as possible. Especially, attention should be paid to the nutritional strategies for very premature infants with hsPDA, or moderate or severe BPD. These measures are of great significance to reduce the incidence of EUGR in very preterm infants with BPD, not small for gestational age.

## Data Availability

The datasets used during the current study are available from the corresponding author upon reasonable request.

## Abbreviations

BPD: Bronchopulmonary dysplasia
CI: Confidence interval
EUGR: Extrauterine growth restriction
FiO_2_: Fraction of inspired oxygen
hsPDA: Hemodynamically significant patent ductus arteriosus
MBDP: Metabolic bone disease of prematurity
NEC: Neonatal necrotizing enterocolitis
NSGA: Non-small for gestational age
OR: Odds ratio
SGA: Small for gestational age
